# Cardio–Renal and Systemic Effects of SGLT2i Dapagliflozin on Short-Term Anthracycline and HER-2-Blocking Agent Therapy-Induced Cardiotoxicity

**DOI:** 10.3390/antiox14050612

**Published:** 2025-05-20

**Authors:** Vincenzo Quagliariello, Annabella Di Mauro, Gerardo Ferrara, Francesca Bruzzese, Giuseppe Palma, Antonio Luciano, Maria Laura Canale, Irma Bisceglia, Martina Iovine, Christian Cadeddu Dessalvi, Carlo Maurea, Matteo Barbato, Alessandro Inno, Massimiliano Berretta, Andrea Paccone, Alfredo Mauriello, Celeste Fonderico, Anna Chiara Maratea, Nicola Maurea

**Affiliations:** 1 Division of Cardiology, Istituto Nazionale Tumori-IRCCS-Fondazione G. Pascale, 80131 Naples, Italy; mart.iovine@gmail.com (M.I.); matteo.barbato@istitutotumori.na.it (M.B.); andrea.paccone@istitutotumori.na.it (A.P.); alfredo.mauriello@istitutotumori.na.it (A.M.); celeste.fonderico@istitutotumori.na.it (C.F.); annachiara.maratea@istitutotumori.na.it (A.C.M.); n.maurea@istitutotumori.na.it (N.M.); 2 Pathology Unit, Istituto Nazionale Tumori-IRCCS-Fondazione G. Pascale, 80131 Naples, Italy; annabella.dimauro@istitutotumori.na.it (A.D.M.); gerardo.ferrara@istitutotumori.na.it (G.F.); 3 Experimental Animal Unit, Istituto Nazionale Tumori-IRCCS-Fondazione G. Pascale, 80131 Naples, Italy; f.bruzzese@istitutotumori.na.it (F.B.); giuseppe.palma@istitutotumori.na.it (G.P.); a.luciano@istitutotumori.na.it (A.L.); 4 U.O.C. Cardiologia, Ospedale Versilia, 55041 Camaiore, Italy; marialaura.canale@uslnordovest.toscana.it; 5 Servizi Cardiologici Integrati, Dipartimento Cardio-Toraco-Vascolare, Azienda Ospedaliera San Camillo Forlanini, 00136 Rome, Italy; irmabisceglia@gmail.com; 6 Department of Medical Sciences and Public Health, University of Cagliari, 09042 Sardinia, Italy; cadedduc@unica.it; 7 UOC Neurology-Stroke Unit, AORN Cardarelli, 80131 Naples, Italy; carlo.maurea@libero.it; 8 Medical Oncology, IRCCS Ospedale Sacro Cuore Don Calabria, 37024 Negrar di Valpolicella, Italy; alessandro.inno@sacrocuore.it; 9 Department of Clinical and Experimental Medicine, University of Messina, 98122 Messina, Italy; berrettama@gmail.com

**Keywords:** cancer, cardio-oncology, cardioprotection, gliflozin, SGLT-2, inflammation, NLRP3, doxorubicin, trastuzumab

## Abstract

Anthracyclines and human epidermal growth factor receptor 2 (HER-2) inhibitors are cornerstone therapies for breast cancer but are associated with significant cardiotoxicity. While sodium–glucose cotransporter 2 (SGLT2) inhibitors such as dapagliflozin have demonstrated cardio–renal protective effects during anthracycline treatment, their efficacy in preventing cardiotoxicity from sequential anthracycline and HER-2 blockade remains poorly understood. This study investigates the cardioprotective role of dapagliflozin in a preclinical model of chemotherapy-induced cardiotoxicity. Female C57Bl/6 mice were divided into four groups and treated for 10 days as follows: (1) a normal control group receiving saline (sham); (2) a model control group receiving doxorubicin (2.17 mg/kg/day for 5 days) followed by HER-2-blocking monoclonal antibody (2.25 mg/kg/day for 5 days); (3) a dapagliflozin-only group (10 mg/kg/day via oral gavage); and (4) a treatment group receiving the combination of doxorubicin, HER-2 inhibitor, and dapagliflozin. Cardiac function was assessed using echocardiography (VEVO 2100). Biomarkers of myocardial injury and inflammation (NLRP3, MyD88, CXCR4, H-FABP, troponin-T, and cytokines) were quantified via ELISA and immunohistochemistry. Circulating markers such as mitofusin-2, cardiac myosin light chain, malondialdehyde (MDA), and 4-hydroxy-2-nonenal (4-HNE) were also measured. Dapagliflozin significantly preserved the ejection fraction and reduced both radial and longitudinal strain impairment in mice treated with the doxorubicin–HER-2 inhibitor combination (*p* < 0.001). Levels of myocardial NLRP3, MyD88, CXCR4, H-FABP, interleukin-1β, and troponin-T were significantly lower in the dapagliflozin-treated group compared to the chemotherapy-only group. Serum markers of oxidative stress and cardiac injury, including mitofusin-2, MDA, 4-HNE, BNP, and high-sensitivity C-reactive protein (hs-CRP), were also reduced by dapagliflozin treatment. Our findings demonstrate that dapagliflozin effectively mitigates early cardiac dysfunction and injury in a preclinical model of sequential doxorubicin and HER-2 inhibitor therapy.

## 1. Introduction

Several anticancer therapies, particularly anthracyclines and targeted agents such as human epidermal growth factor receptor 2 (HER-2) inhibitors (e.g., trastuzumab), have significantly improved survival rates in patients with breast cancer and other malignancies [1]. However, these treatments are frequently associated with cardiotoxic effects that can lead to heart failure, ultimately limiting their long-term clinical utility [2]. The need for effective cardioprotective strategies to counteract these adverse effects is therefore pressing.

Dapagliflozin, a sodium–glucose cotransporter 2 (SGLT2) inhibitor initially developed for the treatment of type 2 diabetes mellitus (T2DM), has demonstrated substantial cardiovascular benefits beyond glycemic control [3]. Clinical studies have shown its efficacy in reducing cardiovascular mortality and hospitalization due to heart failure in both diabetic and non-diabetic patients. More recently, dapagliflozin has emerged as a potential agent for mitigating chemotherapy-induced cardiotoxicity, including in non-diabetic populations [4]. Recent studies have explored the potential cardioprotective benefits of SGLT2 inhibitors, including dapagliflozin, in non-diabetic populations, with growing evidence supporting their use beyond glycemic control [3,4]. Clinical trials such as the DAPA-HF study have demonstrated significant cardiovascular benefits of dapagliflozin in patients with heart failure, regardless of diabetes status. These studies show that dapagliflozin improves heart failure outcomes by reducing hospitalizations and cardiovascular mortality in patients with a reduced ejection fraction, independent of their diabetic condition. Beyond heart failure, SGLT2 inhibitors are increasingly being recognized for their ability to reduce cardiovascular risk factors such as hypertension, kidney dysfunction, and arterial stiffness, making them promising agents in non-diabetic patients at high cardiovascular risk [3,4]. Importantly, emerging preclinical and early clinical studies also suggest that dapagliflozin may mitigate chemotherapy-induced cardiotoxicity, an effect particularly relevant in cancer patients undergoing anthracycline or HER-2-targeted therapies. In these settings, the drug has shown promise in preserving cardiac function and attenuating myocardial damage caused by oxidative stress and inflammation, which are hallmark features of chemotherapy-related cardiotoxicity. This cardioprotective effect in cancer patients has been observed regardless of the presence of diabetes, underscoring the broader therapeutic potential of SGLT2 inhibitors as an adjunct treatment for managing cardiac complications in oncological care [4,5]. Thus, dapagliflozin, initially developed for diabetes management, is now gaining clinical attention for its cardiovascular benefits in non-diabetic populations, including those at risk of chemotherapy-induced cardiotoxicity, with the potential to become a vital therapeutic option in this context [4]. Preclinical evidence suggests that dapagliflozin may offer protection against doxorubicin-induced cardiotoxicity. In animal models, it has been shown to preserve cardiac function—maintaining left ventricular ejection fraction and reducing electrocardiographic alterations—while histological analysis revealed diminished myocardial damage, including reduced sarcomyolysis, inflammatory cell infiltration, and necrosis [5].

Mechanistically, dapagliflozin appears to exert multifaceted cardioprotective effects: it reduces oxidative stress by limiting cytosolic and mitochondrial reactive oxygen species (ROS) production [6]; modulates intracellular calcium dynamics to support contractility; and alleviates endoplasmic reticulum stress. Moreover, it has been shown to inhibit inflammatory signaling pathways, such as nuclear factor-kappa B (NF-κB), which are implicated in the progression of chemotherapy-induced cardiac injury [7].

Despite these encouraging findings, most preclinical studies have primarily addressed the cardioprotective effects of dapagliflozin in the context of anthracycline-only therapy. However, in clinical practice—especially in HER-2-positive breast cancer—patients often receive sequential treatment with anthracyclines followed by HER-2-targeting agents, a regimen associated with cumulative and synergistic cardiotoxicity. To date, no studies have comprehensively explored the biochemical mechanisms underlying dapagliflozin’s cardioprotective effects in this specific sequential treatment model.

In this study, we aimed to investigate for the first time the molecular and functional mechanisms of dapagliflozin-mediated cardioprotection in a preclinical model mimicking the sequential administration of doxorubicin and trastuzumab. By integrating echocardiographic assessment with extensive analysis of inflammatory, oxidative, and apoptotic biomarkers, our work provides novel insights into the pathophysiological basis of chemotherapy-induced cardiotoxicity and supports the potential use of dapagliflozin as a targeted preventive strategy in oncology [8,9].

## 2. Materials and Methods

### 2.1. Preclinical Model of Short-Term Doxorubicin-Trastuzumab Cardiotoxicity

Twenty-four female C57Bl/6 mice (6–7 weeks of age) were purchased from ENVIGO, San Pietro al Natisone (Italy). Mice were housed six per cage and maintained on a 12 h light-12 h dark cycle (lights on at 7:00 a.m.) in a temperature-controlled room (22 ± 2 °C) with ad libitum access to food and water. Preclinical experimental protocols were conducted in accordance with EU Directive 2010/63/EU for animal experiments and Italian D.L.vo 26/2014; they were approved by the Ministry of Health with authorization number 1467/17-PR dated 13 February 2017, and institutional ethics committees, including the Organismo preposto al benessere degli animali (OPBA). Notably, humane endpoints were established in advance, and animals were monitored daily for signs of distress or suffering (e.g., weight loss > 20%, inactivity, abnormal posture, or breathing). Any animal showing signs of severe or irreversible suffering was humanely euthanized, following the ethical protocol. After one week of acclimatization, mice were randomized for weight-adjusted treatment. Mice were divided into four experimental groups (Table 1) (n = 6/group):

Notably, the choice of using six mice per group was based on previous experience with similar short-term cardiotoxicity models, where this number has proven sufficient to detect statistically and biologically relevant differences in key cardiac injury markers. Moreover, we aimed to adhere to the 3Rs principle (Replacement, Reduction, Refinement) in animal research, minimizing the number of animals used while still ensuring scientific validity. Although larger sample sizes (e.g., n = 10) are often used in chronic models, our study focused on acute cardio toxic effects over a 10-day protocol, where variability is lower, and six animals per group provided adequate statistical power, as confirmed by preliminary data and standard deviations observed.

To mimic the sequential therapeutic strategy commonly employed in clinical practice for HER-2-positive breast cancer, mice received doxorubicin for 5 consecutive days followed by HER-2-targeted antibody for an additional 5 days. This 5 + 5 day schedule was chosen to reproduce the clinical sequence of anthracycline administration preceding HER-2 blockade, while allowing the development of early cardiotoxicity in a controlled and reproducible timeframe. Short consecutive-day treatment protocols are widely used in murine models to ensure consistent drug exposure, minimize handling stress, and induce acute myocardial injury suitable for evaluating cardioprotective interventions. The total duration of 10 days was intentionally chosen to assess early cardiac injury and the potential cardioprotective effect of dapagliflozin. This approach aligns with previous studies demonstrating that short-term exposure to anthracyclines can induce significant cardiotoxicity in mice [9,10,11,12].

The dose of doxorubicin (2.17 mg/kg/day for 5 days, cumulative 10.85 mg/kg) was chosen based on previously published murine models of cardiotoxicity, where similar cumulative doses have been shown to induce reproducible myocardial injury without causing excessive mortality [11,12]. This dose is commonly used in acute/subacute cardiotoxicity models, and represents a clinically relevant exposure when adjusted by allometric scaling between mice and humans [12]. The goal was to induce detectable cardiac damage within a short timeframe suitable for evaluating early cardioprotection. For the HER-2-targeted antibody, we used 2.25 mg/kg/day for 5 days, consistent with doses used in previous preclinical studies exploring the cardiac effects of trastuzumab in mice [13,14,15]. This dose has been shown to potentiate cardiac dysfunction, especially when administered following anthracycline treatment, mimicking the clinical scenario of sequential therapy. Importantly, this regimen is sufficient to reveal additive or synergistic cardiotoxic effects without exceeding tolerability in the animal model. Moreover, the choice of dapagliflozin dose and time of therapy was based on recently published studies assessing cardioprotective effects of other drugs, such as ranolazine [16] and empagliflozin [17] against anticancer drug-induced cardiotoxicity. In more detail, the chosen dose of DAPA (10 mg/kg/day via oral gavage) was selected based on a review of several preclinical studies available in the literature [10,11,12,13,14,15,16] and other research involving SGLT2 inhibitors in cardio-oncology [10,17].

### 2.2. Transthoracic Echocardiography and Blood Analysis

A non-invasive transthoracic echocardiography was conducted using the Vevo 2100 high-resolution imaging system (40-MHz transducer; Visual Sonics, Toronto, ON, Canada), consistent with prior studies [18]. Mice were anesthetized with a combination of tiletamine (0.09 mg/g), zolazepam (0.09 mg/g), and 0.01% atropine (0.04 mL/g). Following anesthesia, animals were placed in a supine position on a temperature-controlled surgical table to maintain a rectal temperature of 37 °C, while continuous ECG monitoring was maintained through limb electrodes. Cardiac function was assessed at baseline and at 2 and 10 days of treatment. Left ventricular echocardiography was performed using parasternal long-axis views at a frame rate of 233 Hz. Radial and longitudinal strains were measured due to the complexity of performing apical views in small animal models [19]. This methodology aligns with other studies using VisualSonics (Amsterdam, SK, Canada) Vevo 2100 systems for STE analyses, as previously reported [20]. Image depth, width, and gain settings were adjusted to optimize image quality. End-systolic and end-diastolic dimensions were defined according to ECG phases corresponding to the T and R waves, respectively. M-mode measurements of LV internal dimensions were averaged from 3 to 5 cardiac cycles. Fractional shortening (% FS) was calculated as ((LVID, d − LVID, s)/LVID, d) × 100, and ejection fraction (% EF) was calculated as ((EDvol − ESvol)/EDvol) × 100. Strain was expressed as a percentage, with data imported into the Vevo Strain software package VevoLAB Version 3.0 (FUJIFILM VisualSonics, Toronto, ON, Canada) for detailed analysis. Three consecutive cardiac cycles were analyzed for each mouse, tracing both the endocardium and epicardium. ST-based strain assessments enabled the measurement of 6 myocardial segments per LV view, providing 48 data points per heart. Radial strain (RS) represented the change in myocardial wall thickness, while longitudinal strain (LS) reflected changes in ventricular length. Myocardial deformation rate was also calculated in 1/s. Heart rate during echocardiography was carefully monitored across all experimental groups, with values consistently maintained at approximately 500 bpm (ranging from 490 to 510 bpm), in line with literature [21]. Echocardiographic analyses adhered to “Small Animal Echocardiography using the Vevo^®^ 2100 Imaging System” guidelines and were consistent with previous preclinical studies in cardioncology [22]. Mice were evaluated after 10 days of treatment with DOXO for left ventricular systolic function, heart rate, and cardiac output, as previously described and in accordance with the American Society of Echocardiography’s recommendations [23,24].

### 2.3. Myocardial NLRP-3 and MyD-88 Expression

Following the treatments, hearts were weighed in their entirety. A sample from the left ventricle was then excised, fixed, and embedded in paraffin for histological evaluation (details on the histological effects in the left ventricle are provided in Section 2.6). The remaining heart tissues were processed for quantitative analysis of NLRP-3 and Myd-88. Specifically, the tissues were rapidly frozen using dry ice and stored until homogenization, which was carried out in an appropriate lysis buffer (0.1 M PBS, pH 7.4, supplemented with 1% Triton X-100 and a protease inhibitor cocktail) using a high-intensity ultrasonic liquid processor. The homogenates were centrifuged at 4 °C, and the supernatants were subsequently used for the analysis of NLRP-3 and Myd-88, employing specific ELISA kits for mouse NLRP3 (Mouse NLRP3 ELISA Kit, OKEH05486, Aviva Systems Biology, San Diego, CA, USA.) and MyD88 (Mouse MyD88 ELISA Kit, OKEH03397, Aviva Systems Biology, San Diego, CA, USA.).

### 2.4. Systemic Troponin-T, BNP, H-FABP and hs-CRP Levels

At the conclusion of the treatments, blood samples were collected via cardiac puncture to measure biomarkers associated with cardiotoxicity (Troponin-T, BNP, NT-Pro-BNP) and systemic inflammation (galectin-3 and hs-CRP). Specifically, mouse Troponin-T, BNP, and NT-Pro-BNP levels were assessed using the Mouse Troponin T, cardiac muscle (TNNT2) ELISA kit (CusaBio, Houston, TX, USA), the Mouse BNP ELISA Kit (A77763, Antibodies, Stockholm, Sweden), and the Mouse N-Terminal Pro-Brain Natriuretic Peptide (NT-ProBNP) ELISA Kit (Abbexa, Cambridge, UK), respectively. Galectin-3 was quantified using the Galectin 3 Mouse ELISA Kit (Thermoscientific, Milan, Italy), while hs-CRP was analyzed with the Mouse hs-CRP (high-sensitivity C-reactive protein) ELISA Kit (Elabscience Biotechnology Co., Wuhan, China).

### 2.5. Analysis of Myocardial Lipid Peroxidation and Inflammation

After the treatments, blood samples were obtained via cardiac puncture to measure two biomarkers of lipid peroxidation, such as malondialdehyde (MDA) and 4-hydroxy-2-hexenal (4-HNE). These biomarkers were analyzed using commercially available kits and a spectrophotometer, following the manufacturer’s instructions. Specifically, MDA levels were determined with a Sigma Aldrich kit (MAK085, Milan, Italy), while 4-HNE concentrations were measured using the lipid peroxidation (4-HNE) Assay Kit (ab238538, AbCam, Milan, Italy). Furthermore, the levels of twelve cytokines and growth factors (IL-1α, IL-1β, IL-2, IL-4, IL-6, IL-10, IL-12, IL-17α, IFN-γ, TNF-α, G-CSF, GM-CSF) were assessed using a mouse cytokine Multiplex Assay kit (Qiagen, Germantown, MD, USA, pg/mL) in accordance with literature [25,26].

### 2.6. IHC Staining of CTRD and Pro-Inflammatory Biomarkers

Mouse tissues were fixed in 10% buffered formalin and paraffin embedded with Bioptica automatic processor. To assess histological features Hematoxylin/Eosin (Diapath, Martinengo, Italy) staining was performed according to standard protocol and samples were mounted in Eukitt (Bio-Optica, Milan, Italy). Formalin-fixed, paraffin-embedded (FFPE) heart and kidney sections (4 μm thick) were stained for tissue levels of cardiac and renal NLRP3, MyD-88, IL-1β, IL-6, CXCR4 (and H-FABP and troponin T in myocardial tissue only). The automated VENTANA BenchMark ULTRA method was used for IHC analysis. Tissue slides were deparaffinized using EZ Prep Solution 1X (Ventana Medical Systems, London, UK), heat and vortex mixing [27,28]. Cell Conditioning 1 (CC1, Ventana Medical Systems, UK) was used for antigen retrieval in the presence of heat. Liquid coverslips (LCS) were used to minimize evaporation. Endogenous proteins and peroxides were blocked with Ventana Diluent/Option 1 for 4 min. Primary antibodies were applied to tissue sections and incubated for various durations using previously optimized protocols (Table 2).

In brief, HRP-conjugated secondary antibodies (goat anti-mouse IgG, goat anti-mouse IgM and goat anti-rabbit IgG) were then added to the slides. Visualization of the antibody-antigen reaction was performed using the Ventana Ultraview DAB Universal Detection Kit (Ventana Medical Systems, UK). The NLRP3, MyD-88, IL-1β, IL-6, CXCR4, H-FABP and Troponin T densities (cells/mm^2^) was assessed both by an experienced pathologist and through digital image analysis. Immunostaining values were reported as the percentage of positive cells. The percentage of positive cancer cells in each sample was determined by counting the number of positive cells over the total number of cancer cells in ten non-overlapping fields at 40× magnification. Slides were scanned and photographed using the Aperio Scanscope CS (Aperio Technologies^®^, Vista, CA, USA). Statistical significance calculated using the unpaired *t*-test.

### 2.7. Biomarkers of Damages in Myocardial Tissue

Myocardial tissues were rapidly frozen using dry ice and stored until homogenization, which was carried out in an appropriate lysis buffer (0.1 M PBS, pH 7.4, supplemented with 1% Triton X-100 and a protease inhibitor cocktail) using a high-intensity ultrasonic liquid processor. The homogenates were centrifuged at 4 °C, and the, supernatants were subsequently used for the analysis of several biomarkers of cardiotoxicity, such as Caspase-3 (06-735, Merck, Milan, Italy), Lactate Dehydrogenase (MBS720560; MyBiosource, Milan, Italy), cytochrome C (ab210575; AbCam, Milan, Italy) and Mitofusin-2 (MBS9333527, MyBiosource) through selective ELISA. For intracellular Ca^2+^ quantification, tissue lysate was incubated with 5 µM Fluo-3 AM at 37 °C for 30 min in the dark and then washed three times with PBS (pH 7.4) to remove the excess dye. Fluo-3 chelated with calcium produces fluorescence that was quantified with a spectrofluorometric at excitation and emission wavelengths of 488 nm and 525 nm, respectively.

### 2.8. Statistical Analyses

Continuous data were expressed as the mean ± SD. Nonparametric tests were used both for paired and unpaired comparisons. Repeated-measures ANOVA was used for all baseline to end-of-study comparisons. A *p*-value < 0.05 was considered significant.

## 3. Results

### 3.1. Dapagliflozin Improves EF and Radial/Longitudinal Strain During Anthracycline and HER-2-Blocking Agent Therapy

**Figure 1:**
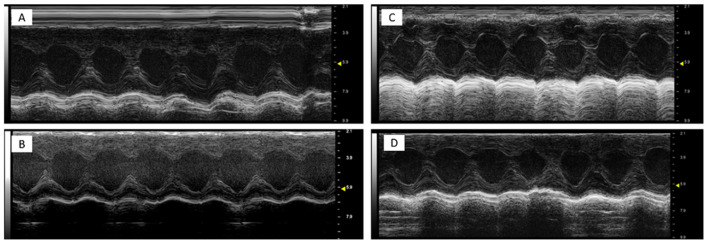
Representative m-mode 2-dimensional images in mice treated with a saline solution (sham, (**A**)), dapagliflozin (**B**), doxorubicin and HER-2-blocking agent (**C**) and dapagliflozin + doxorubicin and HER-2-blocking agent (**D**); (n = 6 for each group). All subfigures were acquired using the same settings with a 10 MHz linear probe. Scale bar: 1 cm.

It was found that DOXO-HER-2 mAbs reduced significantly both EF, FS, radial and longitudinal strain compared to saline group (*p* < 0.0001 for all), indicating cardiotoxicity. Dapagliflozin preserved EF, FS, radial and longitudinal strain reduction during anticancer therapies, suggesting its cardioprotective effects. Representative m-mode images of saline (Figure 1A), DAPA (Figure 1B), DOXO-HER-2 mAbs (Figure 1C) and DAPA + DOXO-HER-2 mAbs (Figure 1D) showed clearly dapagliflozin-related preservation of cardiac function. Heart-related indices, showed in Table 3, were consistent with the representative m-mode 2-dimensional images showed in Figure 1.

### 3.2. Dapagliflozin Inhibits Myocardial Apoptosis, Lipid Peroxidation and Cellular Injury

**Figure 2:**
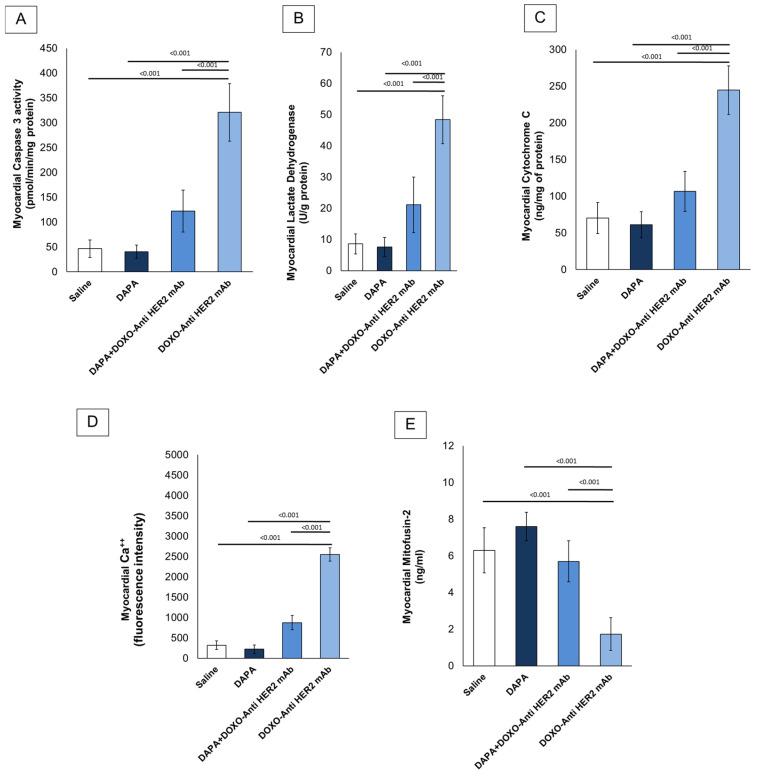
Caspase-3 (**A**), LDH (**B**), cytochrome C (**C**), intracellular Ca^++^ (**D**) and Mitofusin-2 (**E**) expression (pg/mg of protein) in myocardial tissues of mice treated with (saline), DOXO-HER-2-blocking agent, DAPA, and DOXO-HER-2-blocking agent+ DAPA in association (n = 6 for each group).

In line with literature [29], it was found that short-term doxorubicin and HER-2 mAbs therapy in sequential regimen increase myocardial caspase 3, LDH and cytochrome C expression in myocardial tissue of the mice (Figure 2A–C). Notably, although LDH and cytochrome C levels were assessed in whole cardiac homogenates without subcellular fractionation, these measurements were intended to provide an initial overview of myocardial injury and mitochondrial dysfunction. Interpretation should therefore be made cautiously, considering the potential loss of compartmentalization inherent to tissue homogenization. In line with literature, it was found that DOXO-HER-2 mAb increased drastically Ca^++^ in myocardial tissue compared to saline group (*p* < 0.0001); instead, dapagliflozin reduced significantly their levels, in line with literature. It is generally believed that anthracyclines and trastuzumab may reduce mitofusin-2; therefore, we examined its level in myocardial tissue (ng/mL) and found that mitofusin-2 expression was significantly upregulated under dapagliflozin and DOXO-HER-2 mAbs exposure (Figure 2E). Due to the importance of lipid peroxidation in anthracycline and trastumab cardiotoxicity, we investigated the effect of dapagliflozin in MDA and 4-HNA levels in mice. ELISA results showed that MDA (nmol/g protein) and 4-HNA (nmol/g protein) were significantly reduced by DAPA during DOXO-HER-2 mAbs therapy, indicating antioxidant properties and reduction in ferroptosis in mice (Table 4).

The effects of dapagliflozin on the expression of proteins involved in contractile functions were further elucidated. Cardiac myosin light chain (cMLC1), a protein component of the cardiac myosin complex involved in muscle contraction, and Growth differentiation factor type 15 (GDF-15), a member of the TGF-β superfamily, play significant roles in cardiovascular health and are crucial in understanding cardiotoxicity induced by anthracyclines and trastuzumab. As shown in Table 5, their myocardial levels were strongly enhanced after therapy with DOXO-HER-2 mAbs compared to saline (*p* < 0.0001); instead, dapagliflozin was able to reduce significantly their levels, indicating improvement in cardiac contractile functions and injury.

### 3.3. Dapagliflozin Reduces Myocardial Inflammation Through NLRP3 and MyD88

**Figure 3:**
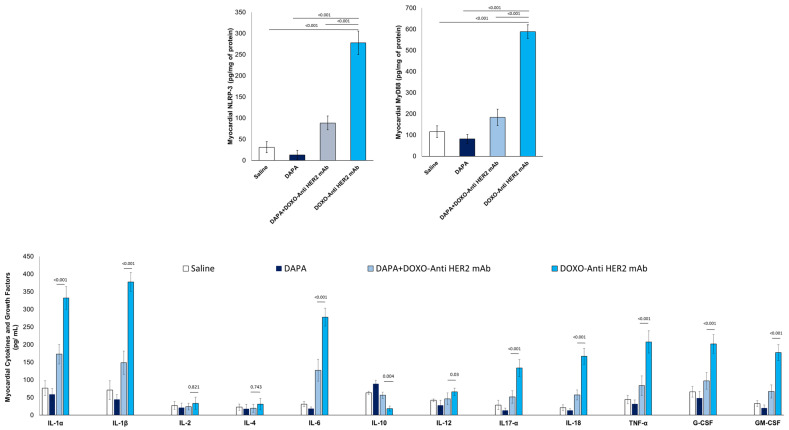
Myocardial NLRP-3, MyD-88 and 12 cytokines in mice treated with (saline), DOXO-HER-2-blocking agent, DAPA 10 mg/kg/day, and DOXO-HER-2-blocking agent+ DAPA in association (n = 6 for each group).

Compared to saline group, DOXO-HER-2 mAbs increased drastically both NLRP3 and MyD-88 myocardial levels (Figure 3), in line with literature [30]; instead, dapagliflozin reduced by 76-81% their expression, indicating anti-inflammatory effects. Since the expression of NLRP3 and MyD-88 are increased, we wandered whether the cytokines and chemokines induced by their pathway exhibit the same trend.

To address this, we used ELISA experiments to detect the expression levels of 12 cytokines and growth factors in myocardial tissue extract of mice exposed to DOXO-HER-2 mAbs alone or associated to DAPA. It was found that IL-1α, IL-1β, IL-6, IL17-α, IL-18, IFN-γ, TNF-α, G-CSF, and GM-CSF were significantly enhanced in DOXO-HER-2 mAbs vs. saline group; instead IL-10 showed an opposite behavior. Notably, dapagliflozin has reversed the trend by reducing the expression of pro-inflammatory cytokines and increasing the levels of the anti-inflammatory cytokine IL-10.

### 3.4. Dapagliflozin Reduces IHC Staining of CXCR4, IL-1, IL-6, HFABP, Troponin-T, NLRP3 and Myd-88 in Myocardial Tissue

**Figure 4:**
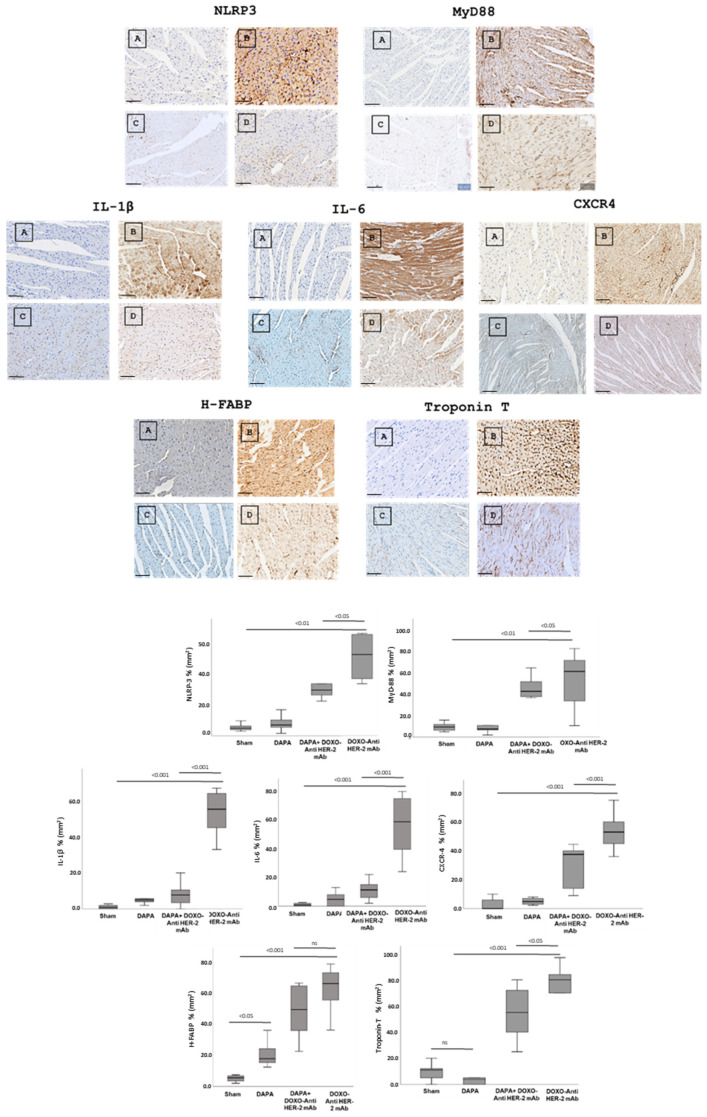
Up, Myocardial NLRP-3, MyD-88, IL-1, IL-6, CXCR4, H-FABP and Troponin-T expression in mice treated with saline (**A**), DOXO-HER-2-blocking agent (**B**), DAPA 10 mg/kg/day (**C**), and DOXO-HER-2-blocking agent+ DAPA in association (**D**) (n = 6 for each group). Scale bar: 5 µm. Down, Quantification of NLRP-3, MyD-88, IL-1, IL-6, CXCR4, H-FABP and Troponin-T cell count in heart belonging to different experimental group (three tissue sections for each heart; six hearts for each group). Global Kruskal–Wallis test with post hoc Mann–Whitney U test was used to analyze the overall difference between groups. Differences were considered significant when *p*-value < 0.05. All data are represented as the mean ± SEM. ns: not significant. Source data are provided as a Source Data file.

In line with quantitative data showed in Figure 3, dapagliflozin has been shown to reduce immunohistochemically (IHC) staining of key inflammatory and myocardial injury markers in myocardial tissue (Figure 4 up). Specifically, dapagliflozin decreased IHC staining of CXCR4, IL-1, IL-6, HFABP, Troponin-T, NLRP3 and MyD-88.

In more detail, myocardial images clearly show that anthracycline and HER-2-blocking agent therapy increases drastically biomarkers of inflammation and heart failure (Figure 4, Down). Dapagliflozin alone did not change significantly their expression confirming its tolerability in preclinical models. Moreover, dapagliflozin associated to chemotherapy reduces significantly tissue expression of CXCR4, IL-1, IL-6, H-FABP, Troponin-T, NLRP3, and MyD-88, demonstrating cardioprotective properties. Quantitative data are in line with the representative IHC images (Figure 4, down), confirming the reduction in the magnitude of cardiotoxicity and inflammation induced by doxorubicin associated to HER-2 mAbs (*p* < 0.001).

### 3.5. Dapagliflozin Reduces IHC Staining of IL-1, IL-6, CXCR4, NLRP3 and Myd-88 in Renal Tissue

**Figure 5:**
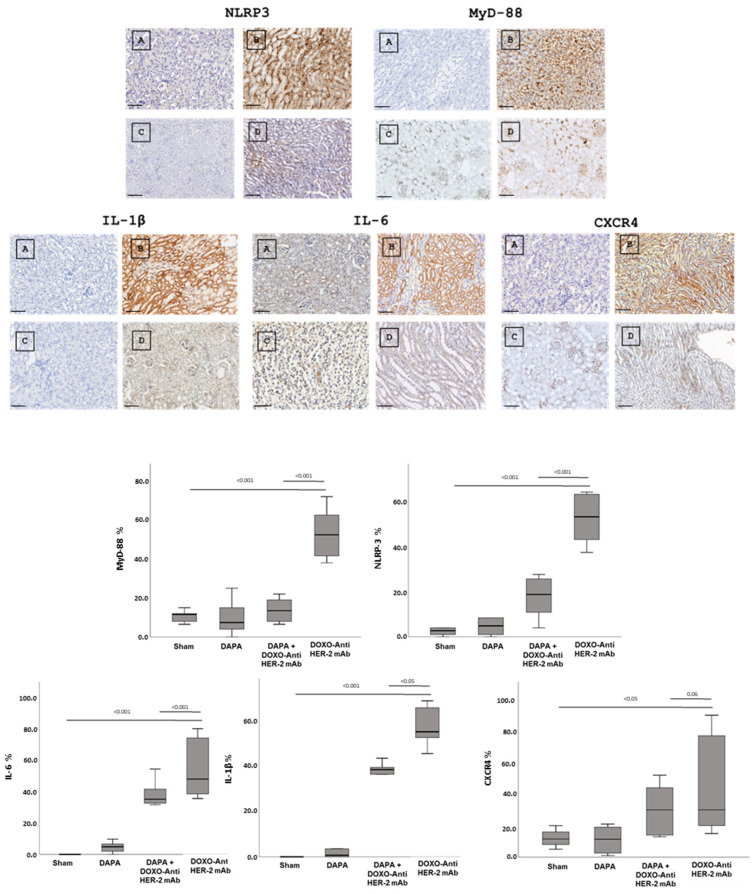
Up, Renal NLRP-3, MyD-88, IL-1, IL-6 and CXCR4 expression in mice treated with saline (**A**), DOXO-HER-2-blocking agent (**B**), DAPA 10 mg/kg/day (**C**), and DOXO-HER-2-blocking agent+ DAPA in association (**D**) (n = 6 for each group). Scale bar: 5 µm. Down, Quantification of NLRP-3, MyD-88, IL-1, IL-6 and CXCR 4 cell count in mice belonging to different experimental group (three tissue sections for each kidney; six kidney for each group). Global Kruskal–Wallis test with post hoc Mann–Whitney U test was used to analyze the overall difference between groups. Differences were considered significant when *p*-value < 0.05. All data are represented as the mean ± SEM. Source data are provided as a Source Data file.

In line with literature [31,32] dapagliflozin treatment led to a significant renal reduction in the IHC staining of IL-1, IL-6, CXCR4 and NLRP3, and MyD-88 (Figure 5, Up). Quantitative data are in line with the representative IHC images (Figure 5, down), confirming the reduction in the magnitude of inflammation and renal injury induced by doxorubicin associated to HER-2 mAbs (*p* < 0.001).

### 3.6. Dapagliflozin Reduces Systemic Levels of CTRD-Related Biomarkers and Inflammation

**Figure 6:**
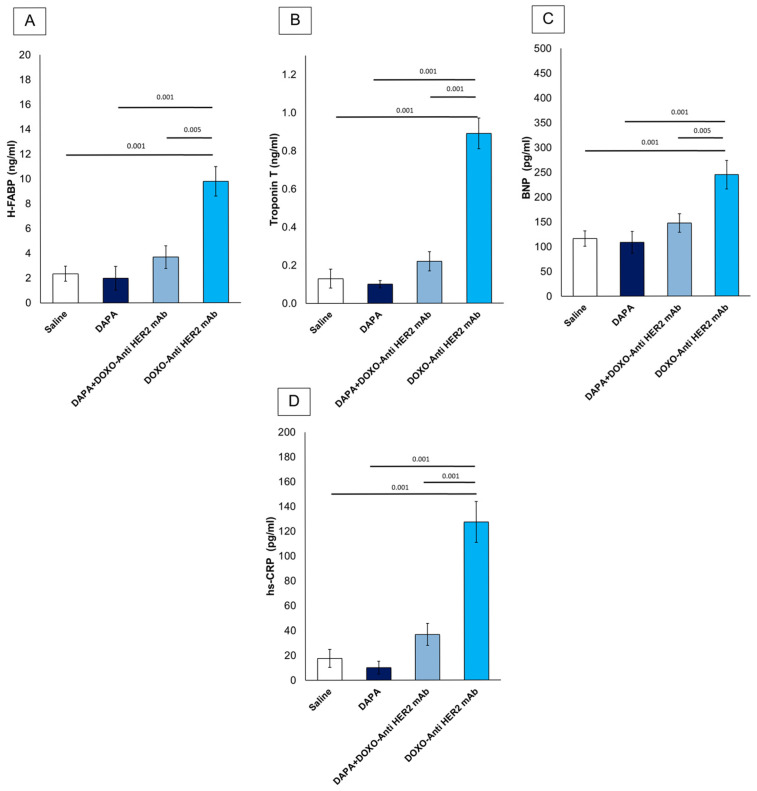
Systemic H-FABP (**A**), Troponin-T (**B**), BNP (**C**) and hs-CRP (**D**) in mice treated with (saline), DOXO-HER-2-blocking agent, DAPA 10 mg/kg/day, and DOXO-HER-2-blocking agent+ DAPA in association (n = 6 for each group).

First, it was studied if dapagliflozin was able to reduce serum levels of H-FABP, Troponin-T, BNP and hs-CRP (Figure 6). All variables were similar between DAPA vs. saline group. Mice treated with anthracyclines and HER-2 mAbs exhibited elevated systemic levels of hs-CRP (+565.5%), H-FABP (+316.17%), BNP (+89.87%) and troponin T (+584.62%) vs. saline group (Figure 6), according to literature [33]. However, the addition of dapagliflozin markedly attenuated these elevations, suggesting its potent role in mitigating CTRD-related damage. Reductions in hs-CRP highlight dapagliflozin’s ability to dampen systemic inflammation, while lower H-FABP, BNP and troponin T levels indicate a protective effect against myocardial injury. These findings underscore the potential of dapagliflozin to modulate inflammatory and cardiotoxic pathways in the context of cancer therapy.

## 4. Discussion

**Figure 7:**
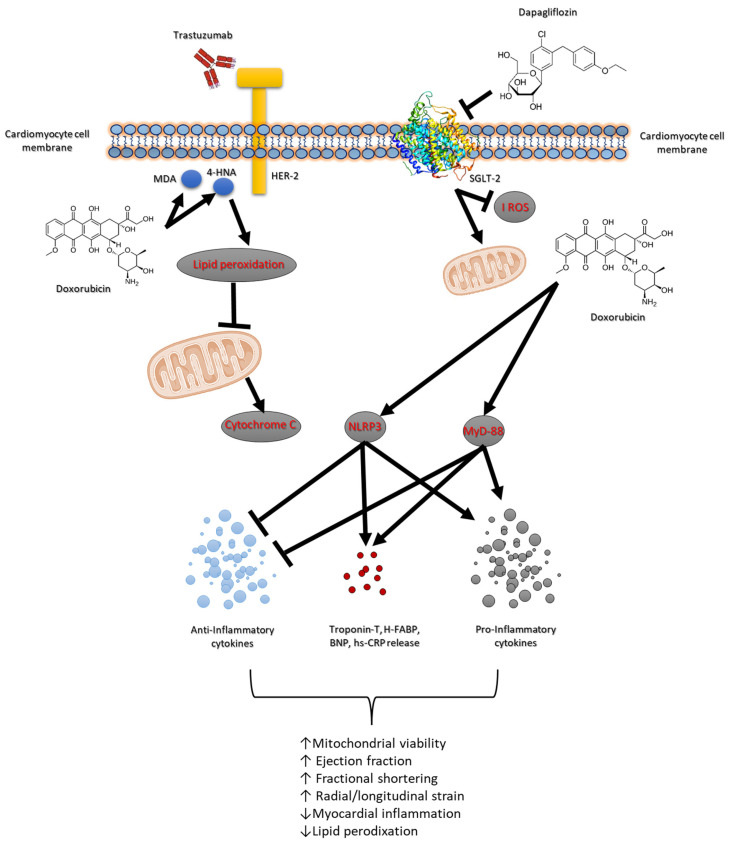
Schematic figure of dapagliflozin-related cardioprotective and antioxidant properties in models of anthracycline and trastuzumab-induced cardiotoxicity.

Doxorubicin and the HER-2-blocking agent trastuzumab are cornerstone therapies in the treatment of breast cancer, significantly improving survival outcomes [34]. However, their combined use is associated with high risk of cardiotoxicity, which can lead to short and long-term cardiovascular complications such as heart failure and reduced cardiac function [35]. The risk of developing cardiac dysfunction increases with cumulative doses, with higher doses leading to a greater likelihood of cardiac injury. Studies have shown that up to 26–48% of patients receiving high cumulative doses of doxorubicin may develop some form of cardiotoxicity, ranging from asymptomatic declines in left ventricular function to severe heart failure [36]. Several factors, including age, pre-existing cardiovascular conditions and co-administration of other cardiotoxic agents (e.g., trastuzumab) further exacerbate the risk. Trastuzumab is highly effective in HER-2-positive breast cancer patients but has been associated with a distinct form of cardiotoxicity known as trastuzumab-induced cardiomyopathy [37,38]. Approximately 15–30% of patients receiving trastuzumab develop some degree of cardiac dysfunction, ranging from asymptomatic reductions in the left ventricular ejection fraction (LVEF) to overt heart failure [37]. Also in this case, some risk factors include older age, pre-existing cardiac disease, concurrent use of other cardiotoxic agents (such as anthracyclines) and longer treatment duration [39]. Although trastuzumab-related cardiotoxicity is generally reversible upon discontinuation of the drug, chronic effects and the development of heart failure remain a concern for cancer survivors [40]. The association of doxorubicin and trastuzumab is increasingly common in the treatment of HER-2-positive breast cancer, significantly elevating the risk of cardiotoxicity [41]. Studies have demonstrated that patients undergoing combination therapy experience a higher incidence of cardiac dysfunction compared to those receiving either agent alone [42]. While doxorubicin contributes primarily to myocyte injury and oxidative stress, trastuzumab amplifies the inflammatory response and impairs myocardial function, increasing the risk of cardiomyopathy. The cumulative cardiotoxic burden of both agents highlights the need for effective interventions to mitigate cardiac damage [43]. A central role in anthracycline and HER-2-blocking agent-mediated cardiotoxicity is played by several key molecular pathways, including the NLRP3, MyD-88 and pro-inflammatory cytokines such as IL-1, IL-6 and chemokine receptor CXCR4 [44,45,46]. These pathways contribute significantly to the pathophysiology of myocardial and renal injury during cancer treatments (Figure 7).

The NLRP3 inflammasome plays a critical role in mediating inflammatory responses in response to cellular stress and injury [47]. In more detail, NLRP3 activation leads to the release of pro-inflammatory cytokines which further propagate inflammation and tissue damage in myocardial tissue. The activation of NLRP3 inflammasome complexes results in the cleavage and secretion of IL-1β and IL-18, which exacerbate myocardial injury and apoptosis [48]. Moreover, NLRP3 promotes oxidative stress through the production of reactive oxygen species (ROS) and lipid peroxidation, thereby amplifying cellular damage in cardiac tissues (Figure 7). MyD-88 is a critical adaptor protein involved in Toll-like receptor (TLR) signaling pathways, which are activated in response to different pathogens and chemotherapy [49]. In line with literature, we have seen that during short-term anthracycline and trastuzumab therapy, the MyD-88-dependent signaling pathway is hyperactivated, leading to the release of inflammatory cytokines such as IL-6. MyD-88 activation amplifies inflammatory signaling cascades that contribute to the upregulation of oxidative stress and pro-apoptotic factors, thereby accelerating myocardial apoptosis and peroxidation [50]. As illustrated in Table 2, MDA and 4-HNA were strongly increased in myocardial tissue of mice treated with anthracycline and trastuzumab, indicating peroxidation.

Moreover, IL-1 and IL-6 are key pro-inflammatory cytokines that play pivotal roles in cardiac inflammation and injury. Both cytokines are involved in the activation of immune cells and the promotion of inflammatory cascades that exacerbate cardiomyocyte apoptosis and dysfunction [51]. IL-1β has been implicated in the regulation of inflammatory pathways that drive myocardial fibrosis and necrosis. IL-6, on the other hand, is a major contributor to chronic inflammation and is linked to the progression of heart failure by promoting hypertrophy and interstitial fibrosis [52]. CXCR4, a chemokine receptor, is crucial for recruiting immune cells to sites of inflammation and tissue injury [53]. In our model of anthracycline and trastuzumab-induced cardiotoxicity (Figure 3), CXCR4 expression is upregulated, facilitating the recruitment of inflammatory cells, which further perpetuates inflammation and myocardial damage [54]. The activation of CXCR4 promotes the release of inflammatory mediators that worsen the overall inflammatory burden, contributing to the exacerbation of tissue injury in the myocardium and kidneys. These biomarkers are associated with inflammation, immune activation, and cardiac damage. CXCR4 plays a crucial role in promoting inflammatory responses and tissue remodeling, while IL-1 and IL-6 are pro-inflammatory cytokines involved in the progression of myocardial injury and heart failure [55]. HFABP is a marker of myocardial injury, and Troponin-T serves as a classic indicator of cardiac damage. NLRP3 and MyD-88 are components of inflammasome pathways that contribute to the amplification of inflammation and tissue damage in response to stress and injury [56]. The reduction in IHC staining for these markers indicates a significant suppression of inflammatory pathways and cellular injury.

Dapagliflozin’s ability to lower the expression of these inflammatory and injury-associated markers suggests its protective role in reducing myocardial damage and preserving cardiac function, particularly during treatments such as anthracycline and trastuzumab that are known to induce cardiotoxicity. This comprehensive attenuation of key markers highlights the potential cardioprotective effects of dapagliflozin in mitigating adverse cardiac outcomes associated with these therapies (Figure 7). The present study highlights the potential of dapagliflozin as a cardio–renal protective agent in primary prevention of anthracycline and trastuzumab-induced cardiotoxicity. Dapagliflozin effectively suppresses the expression of NLRP3, MyD-88, IL-1, IL-6, and CXCR4, thereby reducing the inflammatory signaling pathways responsible for tissue damage. By attenuating these key molecular pathways, dapagliflozin reduces myocardial apoptosis and lipid peroxidation, improving cardiac function. As shown in Table 3, dapagliflozin is able to reduce both MDA, 4HNA and cytochrome c release in myocardial tissue, indicating inhibition of lipid peroxidation and apoptosis. Additionally, dapagliflozin’s ability to lower oxidative stress and decrease several cytokines in an anti-inflammatory phenotype. Moreover, as shown in Figure 6, in renal tissue treated with dapagliflozin, a noticeable decrease in IHC staining for IL-1, IL-6, CXCR4, NLRP3, and MyD-88 was observed. This reduction indicates a suppression of the inflammatory cascade and a reduction in immune cell infiltration, which are critical factors contributing to renal tissue damage. By modulating the expression of these inflammatory markers, dapagliflozin appears to mitigate the severity of renal inflammation and injury, thereby preserving kidney function.

Moreover, the attenuation of these markers suggests that dapagliflozin may play a role in reducing chronic kidney disease progression and preventing complications associated with renal inflammation. The anti-inflammatory effects of dapagliflozin extend beyond its known metabolic benefits, highlighting its potential utility in managing renal-related inflammatory disorders and protecting against renal dysfunction also during anticancer therapies [57,58]. This detailed reduction in IHC staining underscores the renoprotective abilities of dapagliflozin in reduction in renal inflammation and preservation of renal health.

However, several limitations should be acknowledged. Firstly, this study was conducted over a relatively short duration of only 10 days, which may not fully capture the long-term implications of dapagliflozin treatment on cardiac function and its potential cumulative effects. Additionally, while improvements in cardiac function, systemic anti-inflammatory effects, and anti-apoptotic effects were seen, this study lacks long-term monitoring and histological analyses beyond the treatment period. Moreover, the translational relevance of these findings to human patients remains to be established, as murine models may not fully replicate the complexities of human cardiac pathology. Moreover, the quantification of LDH and cytochrome C was performed on total cardiac tissue homogenates without prior subcellular fractionation. As such, the assessment of LDH and cytochrome C levels reflects global tissue content rather than providing specific information about extracellular LDH release or cytochrome C translocation from mitochondria to the cytosol, which are key events in necrosis and apoptosis, respectively. Consequently, the interpretation of these biomarkers as indicators of cardiomyocyte injury or mitochondrial dysfunction must be approached with caution. Future studies should implement subcellular fractionation techniques and analyze extracellular biomarkers to more precisely delineate the mechanisms of chemotherapy-induced cardiac damage and the cardioprotective effects of dapagliflozin.

In our experimental design, we employed a sequential 5-day administration of doxorubicin followed by 5 days of HER-2-targeted antibody treatment to model the clinical practice in HER-2-positive breast cancer patients, where anthracycline-based regimens are typically followed by HER-2 inhibition. The short-term (10-day) protocol was intentionally selected to capture early signs of cardiotoxicity, a critical window during which protective interventions such as dapagliflozin could be evaluated. This approach reflects previous preclinical models where acute/subacute cardiotoxicity was induced using comparable cumulative doses and schedules. Although this abbreviated treatment period does not fully replicate the chronic exposure experienced by patients, it provides a valuable and reproducible framework for mechanistic exploration of cardioprotective strategies in vivo. Future studies employing prolonged regimens will be necessary to confirm these findings in models of chronic cardiotoxicity.

The findings of this study contribute to the growing evidence that dapagliflozin has innovative cardio–renal properties, offering significant therapeutic potential in managing cancer therapy-induced cardiotoxicity.

## 5. Conclusions

This study provides novel evidence of the cardioprotective and anti-inflammatory properties of dapagliflozin in a preclinical model of anthracycline- and HER-2-blocking agent-induced cardiotoxicity. Our results demonstrate that dapagliflozin preserves cardiac function and reduces biomarkers of myocardial injury, oxidative stress, and inflammation in the context of sequential doxorubicin and trastuzumab therapy. These findings suggest that dapagliflozin may serve as a potential pharmacological agent for mitigating chemotherapy-induced cardiotoxicity, particularly in patients undergoing combination therapies known to compromise cardiac health. While our study offers promising insights into the efficacy of dapagliflozin for primary prevention of cardiovascular complications, further clinical investigations are necessary to validate its effectiveness, understand the underlying biochemical mechanisms, and establish optimal therapeutic strategies for its use in oncology patients.

## Figures and Tables

**Table 1:** Experimental groups and treatments to test cardioprotective properties of dapagliflozin against anthracyclines and trastuzumab-induced cardiotoxicity.

Experimental Groups (n = 6)	Treatments
Saline (control)	100 μL saline solution
Doxorubicin-HER-2-blocking agent (Doxo-HER-2 mAb)	Doxorubicin at 2.17 mg/kg/day via intraperitoneal administration (i.p) for 5 days, followed by HER-2 mAb administration at 2.25 mg/kg i.p for an additional 5 days
Dapagliflozin (Dapa)	Dapagliflozin 10 mg/kg/day via oral gavage
Doxorubicin-HER-2-blocking agent + dapagliflozin (Doxo-HER-2 mAb + Dapa)	Doxo-HER-2 mAb + dapagliflozin combination at the same concentrations as each drug administered alone

**Table 2:** Antibodies Used in IHC experiments.

Antibody	Clone	Host	Dilution µg/mL	Code (Company)
**NLRP-3**	MA5-23919	Rat/IgG2a	1:100	Invitrogen (Waltham, MA, USA)
**MYD-88**	(E-11): sc-74532	Mouse/IgG2b	1:150	S.Cruz (Santa Cruz, CA, USA)
**IL-1**	(B-7): sc-9983	Mouse/IgG2b	1:100	S.Cruz (Santa Cruz, CA, USA)
**IL-6**	(E-4): sc-28343	Mouse/IgG2a	1:200	S.Cruz (Santa Cruz, CA, USA)
**CXCR4**	60042-1-Ig	Mouse/IgM	1:150	ProteinTech (Rosemont, IL, USA)
**Troponin-T**	T-C (CT3): sc-20025	Mouse/IgG2a	1:100	S.Cruz (Santa Cruz, CA, USA)
**H-FABP**	328607	Mouse/IgG2a	1:100	Biotechne (Minneapolis, MN, USA)

**Table 3:** Cardiovascular parameters of study groups, such as (saline), DOXO-HER-2-blocking agent, DAPA 10 mg/kg/day, and DOXO-HER-2-blocking agent+ DAPA in association (n = 6 for each group). Statistical analysis was performed using one-way ANOVA followed by Bonferroni’s post hoc test. *^ns^* not significant, *^b^* *p* < 0.005, *^c^* *p* < 0.001 comparing saline to DOXO-HER-2 mAb mice, *^A^* *p* < 0.01, *^B^* *p* < 0.005, *^C^* *p* < 0.001 comparing DOXO-HER-2 mAb mice to DAPA + DOXO-HER-2 mAb.

Heart-Related Indices	Sham (Saline)	DOXO-HER-2 mAb	DAPA	DAPA + DOXO-HER-2 mAb
**IVS; d-D (mm)**	0.54 ± 0.09	0.61 ± 0.09 *^ns^*	0.47 ± 0.13	0.56 ± 0.12 *^ns^*
**LVID; d-D (mm)**	1.92 ± 0.21	3.1± 0.18 *^c^*	1.87 ± 0.11	2.37 ± 0.28 *^C^*
**LVPW; d-D (mm)**	0.57 ± 0.12	0.6 ± 0.08 *^ns^*	0.52 ± 0.07	0.58 ± 0.12 *^ns^*
**LV Mass (mg)**	48.8 ± 2.8	58.8 ± 1.9 *^c^*	44.1 ± 1.8	49.7 ± 1.66 *^B^*
**LVID; s-D (mm)**	1.37 ± 0.4	1.82 ± 0.33 *^b^*	1.31 ± 0.08	1.48 ± 0.17 *^A^*
**EF (%)**	94.28 ± 1.8	60.2 ± 2.6 *^c^*	95.71 ± 1.3	90.8 ± 2.32 *^C^*
**FS (%)**	66.35 ± 2.9	38.1 ± 2.12 *^c^*	67.1 ± 2.41	61.2 ± 2.63 *^C^*
**Radial Strain (Pk%)**	38.7 ± 2.2	9.7 ± 1.7 *^c^*	39.6 ± 1.8	30.8 ± 2.88 *^C^*
**Longitudinal Strain (Pk%)**	−21.8 ± 1.32	−10.2 ± 1.9 *^c^*	−22.4 ± 3.8	−19.3 ± 3.2 *^B^*
**Heart weight (g)**	0.14 ± 0.07	0.27 ± 1.3 *^ns^*	0.13 ± 0.04	0.16 ± 0.08 *^ns^*

**Table 4:** Myocardial MDA and 4-HNA levels (nmol/g of protein) in myocardial tissues of mice treated with (saline), DOXO-HER-2-blocking agent, DAPA 10 mg/kg/day, and DOXO-HER-2-blocking agent+ DAPA in association (n = 6 for each group). Statistical analysis was performed using one-way ANOVA followed by Bonferroni’s post hoc test. *^c^* *p* < 0.001 comparing saline to DOXO-HER-2 mAb mice, *^B^* *p* < 0.005, *^C^* *p* < 0.001 comparing DOXO-HER-2 mAb mice to DAPA + DOXO-HER-2 mAb.

Lipid Peroxidation Products	Sham (Saline)	DOXO-HER-2 mAb	DAPA	DAPA + DOXO-HER-2 mAb
**MDA (nmol/g protein)**	0.032 ± 0.008	0.241 ± 0.03 *^c^*	0.021 ± 0.003	0.059 ± 0.09 *^B^*
**4-HNA (nmol/g protein)**	0.052 ± 0.003	0.32 ± 0.07 *^c^*	0.041 ± 0.006	0.067 ± 0.07 *^C^*

**Table 5:** Myocardial Cardiac myosin light chain (cMLC1) 1 (ng/mL) and Growth differentiation factor type 15 (GDF-15) (ng/mL) in mice treated with (saline), DOXO-HER-2-blocking agent, DAPA 10 mg/kg/day, and DOXO-HER-2-blocking agent+ DAPA in association (n = 6 for each group). Statistical analysis was performed using one-way ANOVA followed by Bonferroni’s post hoc test., *^c^* *p* < 0.001 comparing saline to DOXO-HER-2 mAb mice, *^C^* *p* < 0.001 comparing DOXO-HER-2 mAb mice to DAPA + DOXO-HER-2 mAb.

Novel Biomarkers of Cardiovascular Injury	Sham (Saline)	DOXO-HER-2 mAb	DAPA	DAPA + DOXO-HER-2 mAb
**Cardiac myosin light chain (cMLC1) 1 (ng/mL)**	2.23 ± 0.04	5.44 ± 0.09 *^c^*	2.18 ± 0.06	3.14 ± 0.03 *^C^*
**Growth differentiation factor type 15 (GDF-15) (ng/mL)**	1.25 ± 0.06	4.33 ± 0.13 *^c^*	1.13 ± 0.11	1.85 ± 0.15 *^C^*

## Data Availability

The datasets analyzed for this study can be found in the Zenodo repository (https://zenodo.org/records/14809939) accessed on 20 March 2025.

## Abbreviations

The following abbreviations are used in this manuscript:

DOXO	Doxorubicin
HER-2	Human Epidermal Growth Factor Receptor 2
SGLT2	Sodium–Glucose Cotransporter 2
T2DM	Type 2 Diabetes Mellitus
NF-κB	Nuclear Factor Kappa-Light-Chain-Enhancer of Activated B Cells
mAb	Monoclonal Antibody
DAPA	Dapagliflozin
LV	Left Ventricle
ECG	Electrocardiogram
FS	Fractional Shortening
EF	Ejection Fraction
RS	Radial Strain
LS	Longitudinal Strain
STE	Speckle-Tracking Echocardiography
NLRP3	NOD-, LRR- and Pyrin Domain-Containing Protein 3
MyD88	Myeloid Differentiation Primary Response 88
ELISA	Enzyme-Linked Immunosorbent Assay
BNP	Brain Natriuretic Peptide
NT-ProBNP	N-Terminal Pro-Brain Natriuretic Peptide
hs-CRP	High-Sensitivity C-Reactive Protein
MDA	Malondialdehyde
4-HNE	4-Hydroxy-2-Nonenal
IL	Interleukin
IL-1α	Interleukin-1 Alpha
IL-1β	Interleukin-1 Beta
IL-2	Interleukin-2
IL-4	Interleukin-4
IL-6	Interleukin-6
IL-10	Interleukin-10
IL-12	Interleukin-12
IL-17α	Interleukin-17 Alpha
IFN-γ	Interferon-Gamma
TNF-α	Tumor Necrosis Factor Alpha
G-CSF	Granulocyte Colony-Stimulating Factor
GM-CSF	Granulocyte-Macrophage Colony-Stimulating Factor
CTRD	Chemotherapy-Related Cardiotoxicity
CXCR4	C-X-C Chemokine Receptor Type 4
H-FABP	Heart-Type Fatty Acid-Binding Protein
FFPE	Formalin-Fixed, Paraffin-Embedded
